# Radiotherapy in metastatic bladder cancer

**DOI:** 10.1007/s00345-023-04744-x

**Published:** 2024-01-20

**Authors:** Sophie Ashley, Ananya Choudhury, Peter Hoskin, YeePei Song, Priyamvada Maitre

**Affiliations:** 1 The Christie NHS Foundation Trust, Manchester, United Kingdom; 2 Division of Cancer Sciences, University of Manchester, Manchester, United Kingdom; 3 Manchester Biomedical Research Centre, Manchester, United Kingdom; 4 Mount Vernon Cancer Centre, Northwood, United Kingdom; 5 Present Address: Department of Radiation Oncology, Tata Memorial Hospital and Advanced Centre for Treatment Research and Education in Cancer (ACTREC), Homi Bhabha National Institute (HBNI), Ernest Borges Road, Parel, Mumbai, India

**Keywords:** Metastatic bladder cancer, Oligometastatic bladder cancer, Bladder radiotherapy, Palliative radiotherapy

## Abstract

**Purpose:**

To review available and emerging evidence of radiotherapy for symptom management and disease control in metastatic bladder cancer.

**Methods:**

A literature search and subsequent cross-referencing were carried out for articles in the PubMed and Scopus databases using terms ‘radiotherapy’ OR ‘palliative radiation therapy’ with ‘metastatic bladder cancer’ OR ‘advanced bladder cancer’ between 1990 and 2023, excluding articles with no English translation.

**Results:**

Palliative radiotherapy is an effective and accessible treatment for the alleviation of haematuria and pain due to the primary and metastatic disease. With growing recognition of oligometastatic disease state at diagnosis, response, or progression, radiotherapy can consolidate response by ablating residual or resistant lesions. Experience with other primary cancers supports positive impact of radiotherapy on disease control, quality of life, and survival in oligometastatic stage, without significant adverse effects. Alongside immune checkpoint inhibitors, fibroblast growth receptor inhibitors, and antibody–drug conjugates, the immunomodulatory potential of radiotherapy is being explored in combination with these systemic therapies for metastatic bladder cancer.

**Conclusion:**

Radiotherapy is an effective, safe, and accessible treatment modality for palliation as well as disease control in various clinical settings of metastatic bladder cancer. Its role in oligometastatic stage in combination with systemic therapy is expected to expand with emerging evidence.

## Introduction

Bladder cancer causes nearly 200,000 deaths each year globally [1]. About 10–15% patients with diagnosed with bladder cancer have metastatic spread at presentation [1, 2]. In the UK, bladder cancer causes about 5000 deaths annually [3]. Lower socioeconomic strata are disproportionately affected, with 5-year overall survival of 46% versus 56% in the highest socioeconomic group [4]. Metastatic bladder cancer (MetBC) includes involvement of lymph nodes beyond the pelvis, or other visceral organs. The prognosis at this stage is dismal, with less than 10% patients surviving at five years from the diagnosis [5]. It also causes considerable morbidity, with urinary symptoms of recurrent or intractable haematuria, pelvic pain, dysuria, and obstructive renal failure. First-line treatment is platinum-based chemotherapy, cisplatin and gemcitabine combination recommended for patients with good renal function (glomerular filtration rate, GFR < 60 mL/min) and performance status of 0–1. Carboplatin is an accepted compromise for those with impaired renal function or worse performance status [3]. Five-year survival of up to 15% can be expected with cisplatin-based chemotherapy [6]. If the disease responds or stabilises with first-line chemotherapy, further maintenance immunotherapy using avelumab has shown to prolong overall survival [7]. First-line immunotherapy as a single agent or within chemotherapy combination has not shown survival benefit [8, 9].

Radiotherapy is a curative treatment for localised and node-positive muscle-invasive bladder cancer, using high-energy ionising radiation to induce cancer cell death [3, 10–13]. For MetBC, it is an effective treatment for palliation of symptoms such as pelvic pain and haematuria from advanced primary disease, usually requiring 1–5 fractions [14]. Radiotherapy for alleviating painful or symptomatic metastases is a non-invasive, inexpensive, and accessible palliative treatment. Technological advances such as stereotactic ablative radiotherapy (SABR) have made it possible to accurately and safely irradiate tumour lesions to high dose within a few fractions. As the evidence for safety and effectiveness of SABR for metastases has emerged, there is growing interest in pushing the boundaries of curative treatment. Patients with a small number of discrete metastases (oligometastases) are being recognised as a separate group to those presenting with widespread disease [15]. Simultaneously, survival benefit with radiotherapy to the primary tumour has been observed in metastatic stage in malignancies such as prostate cancer [16]. This review considers the role of radiotherapy in symptom management and overall disease control in metastatic bladder cancer. A literature search and subsequent cross-referencing was undertaken for articles in the Pubmed and Scopus databases using terms ‘radiotherapy’ OR ‘palliative radiation therapy’ with ‘metastatic bladder cancer’ OR ‘advanced bladder cancer’ between 1990 and 2023, excluding articles with no English translation.

## Radiotherapy for symptom palliation

Effectiveness of radiotherapy for relief of urinary symptoms caused by locally advanced cancer is well established. Table 1 summarises the evidence for palliative radiotherapy for bladder cancer [14, 17–23]. The earliest of these studies suggests that hypofractionated radiotherapy provides almost universal relief in haematuria, with complete symptom control in over half of the patients [17]. The MRC-BA09 randomised trial established the efficacy of 3-fraction schedule for palliative bladder radiotherapy [14]. For patients without metastatic cancer but considered unsuitable for radical treatment, hypofractionated radiotherapy of 36 Gy in six weekly fractions achieved disease control in 92% of the assessable patients at three months [19]. Cumulative rate of local progression post-radiotherapy was low, being 7% at one year and 17% at 2 years. Grade 3 urinary toxicity and gastrointestinal toxicity were seen in 18% and 4% patients, respectively, during treatment, but at 6 months only 6.5% patients reported any grade 3 toxicity. A more recent study observed clinical outcomes with commonly used schedules of palliative radiotherapy, such as 8 Gy in 1 fraction, 21 Gy in 3 fractions, 20 Gy in 5 fractions, 36 Gy in 6 fractions, or 27.5–30 Gy in 8–10 fractions [20]. Symptomatic improvement such as resolution of haematuria (in 54% patients), dysuria or urinary frequency (57%), and pain control (48%) was observed. Patients treated with 36 Gy dose had better overall survival on univariable analysis (hazard ratio, HR 0.45, *p* < 0.001), which did not remain significant in multivariable analysis (HR 0.81, *p* = 0.3) suggesting potential confounding by patient selection for different schedules. More importantly, about a quarter of patients treated with palliative bladder radiotherapy either discontinued treatment or died with a month of starting radiotherapy. This study emphasised the importance of patient selection for palliative radiotherapy based on performance status, disease stage, severity of symptoms, and expected prognosis, to optimise the palliative benefit.

**Table 1:** Studies of palliative radiotherapy for bladder cancer

Author, year	Type of study	Number of patients (with metastases)	Dose (range)	Clinical outcome	Adverse effects/remarks
McLaren et al., Radiother Oncol 1997 [17]	Retrospective, single centre	65 (04)	30-36 Gy in 5–6 fractions weekly	Complete palliation of haematuria in 92% Complete palliation of symptoms in 51% 71% experienced symptomatic improvement within 1 month	15% had worsening of dysuria and frequency 11% had persistent bowel symptoms
Jose et al., Clin Oncol 1999 [18]	Prospective single centre	65 (06)	12 Gy in 2 fractions, 18 Gy in 3 fractions, 24 Gy in 4 fractions, 30-36 Gy in 5–6 fractions weekly	62% complete response on cystoscopy 25% local control Median survival 35 weeks 2-year actuarial survival 21%	Acute urinary toxicity grade 3 (frequency) 10.8%, grade 4 (obstruction) 1.5% Late urinary toxicity grade 3 9%, grade 4 1.5%
Duchesne et al., IJROBP 2000 [14]	Prospective multicentric randomised trial	272 (40)	35 Gy in 10 fractions daily vs 21 Gy in 3 fractions	68% had symptomatic improvement (71% for 35 Gy, 64% for 21 Gy) Survival similar between schedules (HR 0.99, *p* = 0.93)	Median duration of symptom control 9 months No difference in efficacy or toxicity between the two schedules
Dirix et al., Support Care Cancer 2016 [21]	Prospective, multicentric	44	34.5 Gy in 6 fractions weekly	91% were hematuria free at median 10 months Mean hematuria-free survival 13 months	9% acute, 19% late ≥ grade 3 urinary toxicity
Hafeez et al., IJROBP 2017 [19]	Prospective single centre	55 (02)	36 Gy in 6 fractions weekly	Local control 93% at 1 year, 83% at 2 years 1-year OS 63%	Acute grade 3 toxicity 18% urinary, 4% gastrointestinal Grade ≥ 3 late toxicity (any) 6.5% at 6 months, 4.3% at 12 months No grade 4 acute or late toxicity
Ali et al., IJROBP 2019 [20]	Retrospective, multicentric	241 (100)	8 Gy in 1 fraction, 21 Gy in 3 fractions, 20 Gy in 5 fractions, 36 Gy in 6 fractions, or 27.5–30 Gy in 8 to 10 fractions	Median OS 153 days Symptomatic improvement in 53% within 6 weeks of radiotherapy	25% of patients either did not complete radiotherapy or died within 30 days of starting treatment 30-day mortality 18% 14% did not complete planned RT
Tey et al., In Vivo 2019 [22]	Retrospective, multicentric	58 (18)	8 Gy in 1fraction to 40 Gy in 16 fractions	Hematuria improved in 67% patients Median duration of response 3.7 months Median OS 5.6 months	Acute toxicity 1.7% nausea grade 3, 3.5% diarrhoea grade 1–2 No grade ≥ 3 late toxicity Better control of hematuria with ≥ 36 Gy biologically effective dose
Ogita et al., Sci Rep 2021 [23]	Retrospective, multicentric	53 with any pelvic cancer (22 with bladder primary)	30 Gy in 10 fractions, 20 Gy in 5 fractions, 36 Gy in 12 fractions	76% patients became free of gross hematuria Median duration of hematuria control 4.3 months 1-year OS 35%	No grade ≥ 3 acute toxicity, grade 2 5.6% diarrhoea, 1.8% proctitis Better control of hematuria with ≥ 36 Gy biologically effective dose

Reliable comparison of efficacy among various palliative schedules is difficult due to small patient cohorts. Biologically effective dose (BED) of ≥ 36 Gy (*a*/*b* = 10) was associated with longer and better control of haematuria in two studies [22, 23]. Haematuria in patients treated with BED ≥ 36 Gy responded better than < 36 Gy (77% vs 61%), remained controlled for longer (median control 8.4 months vs 0.7 months, HR 0.39, *p* = 0.02), and recurred less (HR 5.76 for < 36 Gy vs > 36 Gy, *p* = 0.01). However, no difference was observed on overall survival [20, 22]. The schedule of 36 Gy in six fractions once-weekly has shown durable symptom palliation in 50–90% patients as well as local disease control up to 90% at one year, with about 38–42% grade 2 and 4–20% grade 3 acute toxicity [17, 19, 21] even if it has never been compared prospectively to other established radiotherapy schedules. For patients with life expectancy of less than six months, one to three fractions appear convenient to provide adequate symptom relief. A higher dose may be preferable for patients with lower disease burden, better performance status and relatively longer life expectancy, although MRC BA06 confirmed similar clinical outcomes of 21 Gy in 3 fractions to 35 Gy in 10 fractions.

## Metastases-directed radiotherapy for oligometastatic bladder cancer

MetBC includes loco-regional and distant metastases of varying disease burden. Oligometastatic stage has been suggested as a transition between limited and extensive spread, which has been further refined into oligoprogression (for metastatic progression), oligopersistence (residual during treatment), or oligorecurrence (during a treatment free interval) [15]. Early clinical trials suggest that targeting oligometastases with SABR for selected patients prolongs disease control with few adverse effects. The prospective trials recruited oligometastases of mixed primary origin, of which bladder cancer comprised a small proportion [24–26]. Specifically for bladder or urothelial cancer, a few retrospective studies (Table 2) have reported clinical outcomes with SABR for oligometastases [27–32]. Lymph nodes were the most commonly treated lesions in these case series [27–29, 31]. A retrospective case series of 91 patients with up to 5 metastases reported improved overall survival (HR 0.48, *p* = 0.026) and progression free survival at 6 months (HR 0.57, *p* = 0.083) for those who received consolidative radiotherapy to the bladder and residual metastases compared with those in an observation group, with local nodes treated most commonly (64%) [29]. Another multicentre retrospective analysis of 61 patients with ≤ 5 metastases at diagnosis reported on outcomes after SABR to total 82 lesions, commonest being lung (40%) [30]. After a median follow-up of 17 months, local control of 92% at 1 year and 89% at 2 years was observed. Patients benefitted from systemic therapy before SABR (local control HR 2.62, p = 0.034) and higher total dose (OS HR 0.93, *p* = 0.003), and number of metastases was predictive for progression-free survival (HR 2.65, p = 0.008). Importantly, consolidative radiotherapy was overall well tolerated in these studies, with 0–3% grade 3 toxicity and no grade 4 toxicity. To note, two cohorts had local treatment to the bladder primary in addition to metastases-directed radiotherapy [29, 32]. First study had 15 patients treated with primary cystectomy followed by metastatic recurrence and first-line chemotherapy, with radiotherapy to oligoresidual lesions [32], and the second study had 91 patients treated with radiotherapy to bladder and oligometastases [29]. Median progression-free survival in these cohorts was 13–15 months, in contrast to 3–6 months for other cohorts of metastases-directed radiotherapy. With the caveats of heterogeneous inclusion criteria and treatment approaches, treatment of both the oligometastases and the bladder primary may result in more durable disease control.

**Table 2:** Studies of stereotactic ablative radiotherapy for oligometastatic urothelial cancer

Author, year	Type of study	Primary tumour	Inclusion criteria	No. of patients (n)	Dose (range)	Treatment	Adverse effects	Survival outcomes
Shah et al., Clin Genitourin Cancer 2017 [32]	Retrospective, single centre	Urothelial bladder cancer	Oligoresidual metastases after at least partial response to first-line chemotherapy (for metastatic recurrence post-cystectomy)	22		Radiotherapy after partial response to chemotherapy		Median PFS 13 months Median OS 29 months
Leonetti et al., Int J Urol 2018 [27]	Retrospective, single centre	Urothelial cancer (including upper tract)	≤ 3 node-only metastases	7 (14 nodal lesions)	Mean 32 Gy (range 25–40) in 5 fractions	Node-directed SABR	–	Median PFS 2.9 months Median OS 14.9 months
Augugliaro et al., Neoplasma 2019 [28]	Retrospective, single centre	Urothelial bladder cancer	≤ 5 metastases (node, bone, lung, or local recurrence)	13 (21 lesions)	Median 25 Gy (20–36) in 5 fractions	Metastasis-directed RT	7.7% acute grade 1	Median radiological PFS 4.2 months
Francolini et al., Cancer Treat Res Commun 2019 [31]	Retrospective, single centre	Urothelial cancer	≤ 3 metastases (or local recurrence)	19 (25 lesions)	18-60 Gy in 1–8 fractions	Metastases-directed SABR	Acute grade 1 nausea, asthenia, dysphagia no late toxicity	Local control 68% Median PFS 5.6 months Mean OS 13.8 months
Franzese C et al., Clin Oncol 2020 [30]	Retrospective, multicentric	Urothelial cancer (including upper tract)	≤ 5 metastases	61 (82 lesions)	Median 45 Gy (18–70) in 1–10 fractions	Metastasis-directed SABR	6.56% acute grade 1 1.64% late grade 1 No grade ≥ 2	Local control 1-year 92%, 2-year 89% PFS 1-year 48%, 2-year 38% Median OS 25.6 months
Aboudaram et al., Cancers 2023 [29]	Retrospective, multicentric	Urothelial bladder cancer	≤ 5 residual metastases after first-line therapy	91 (treatment = 51, observation = 40)	Median EQD2 53 Gy (45–132) to metastases, 64 Gy (45–66) to bladder	Radiotherapy to bladder and metastases	2.6% grade 3, no grade 4–5	Median PFS 14.8 vs 9.7 months (HR = 0.57, *p* = 0.082) Median OS 29.7 vs 19.7 months (HR = 0.48, *p* = 0.026)

Recently, oligometastatic bladder cancer (OMBC) was formally recognised by a consensus of the European academic oncologists and urologists [33]. The consensus definition for OMBC was based on feasibility of SABR, as “a maximum of three metastatic sites, all resectable or amenable to stereotactic therapy”. A few important aspects of this definition relevant to clinical practice are as follows:

- Applicability—The same definition has been agreed upon for all three OMBC subgroups, i.e. synchronous OMBC, metachronous oligorecurrence, and metachronous oligoprogression.
- Staging imaging—In contrast to traditional imaging-based staging, no consensus was reached on the imaging modality to be used for staging OMBC. Utility of fluorodeoxyglucose (FDG) positron emission tomography/computed tomography (PET/CT) is yet to be established pending prospective evidence [34], and contrast-enhanced CT and/or magnetic resonance imaging (MRI) remain the diagnostic standard.
- Organ sites—Pelvic lymph nodes were not specifically recognised as a separate organ site for metastasis. This aligns with the American Joint Committee on Cancer (AJCC) staging, where pelvic nodal positivity is staged as non-metastatic. Extra-pelvic lymph nodes are considered metastatic by the AJCC as well as the OMBC consensus definition.
- Patient selection—Favourable response to systemic treatment is a key criterion to be considered for metastasis-directed therapy, emphasising the importance of tumour biology and systemic control before the feasibility of local treatment.

Hopefully, this definition would provide a common standard for future research in OMBC. It also leaves the door open for future advancements in radiotherapy technology, to push the limits of number of metastatic sites which can be treated safely. As the ablative radiotherapy evolves in OMBC, exploring the utility of metastasectomy vis-à-vis radiotherapy for disease control would likely be of interest for the urologists [35].

## Bladder radiotherapy for metastatic bladder cancer

Apart from symptom control, radiotherapy to the bladder primary is being explored for benefits of disease control and survival. This hypothesis is being extrapolated from recent evidence from other cancers. A recent meta-analysis of about 5000 patients with metastatic cancer, who received local treatment (surgery or radiotherapy) for the primary disease site, observed that radiotherapy to the primary was associated with significant improvement in overall survival (HR 0.67, 95% CI 0.52–0.85) in patients with low metastatic burden [36]. This benefit was not observed for high metastatic burden, nor with surgery for oligometastatic disease. The strongest evidence for survival benefit of radiotherapy in oligometastatic cancer is in prostate cancer and nasopharyngeal cancer [16, 37, 38]. Proposed biological rationale are reduced seeding of metastases by irradiating the prostate primary, and improved local control leading to reduced morbidity and mortality for nasopharyngeal cancer [36]. It is yet unclear if any or both hypotheses would be applicable to bladder cancer.

Morbidity of locally advanced disease in MetBC is well known, with neuromuscular and visceral progression within the pelvis causing intractable pain, bleeding, fistulae, and sepsis leading to eventual death. Local disease control by early radiotherapy to the bladder in OMBC may prevent this mortality. Studies addressing this question *(*Table 3*)* are mainly retrospective, single or multi-institutional series [39–41]. While the largest cohorts are from National Cancer Database (NCDB), their results are hampered by inherent shortcomings of this database such as selection bias and inability to extract treatment details of radiotherapy or chemotherapy. There are also a few case series of cystectomy as a consolidative local treatment instead of radiotherapy for patients with favourable response after systemic therapy [39, 42, 43]. A systematic review observed 5-year OS of 28% with a combined approach of complete surgical removal of primary as well as oligometastases after partial response with chemotherapy [44]. The role of these local treatment modalities for de novo or post-chemotherapy OMBC will need to be prospectively studied to reliably determine a clinical benefit.

**Table 3:** Local treatment for bladder primary in metastatic bladder cancer

Author, year	Type of study	Inclusion criteria	*N*	Local treatment	Survival outcomes	Adverse effects
Fischer-Valuck et al., Eur Urol Oncol 2022 [40]	Retrospective data from National Cancer Database	Newly diagnosed MBC treated with first-line chemotherapy	4459 (bladder radiotherapy yes = 337, no = 4122)	Bladder radiotherapy (median dose 57.6 Gy)	Median OS 13.8 months vs 8.5 months (*p* < 0.0001) HR 0.70 (95% CI 0.62–0.79, *p* < 0.0001)	Not reported
Seisen et al., J Clin Oncol 2016 [39]	Retrospective data from National Cancer Database	MBC treated with first-line chemotherapy	3753 (local treatment yes = 297, no = 3456)	Radical cystectomy or bladder radiotherapy ≥ 50 Gy	Median OS 15 months vs 10 months (*p* < 0.001) HR 0.56 (95% CI 0.48–0.65, *p* < 0.001)	Not reported
Ho et al., Urol Oncol 2016 [43]	Retrospective, single centre	Bladder cancer with pelvic/retroperitoneal lymph nodal involvement, no visceral metastases, treated with first-line chemotherapy	55	Consolidative lymph node dissection with cystectomy	Median CSS 25.7 months 5-year PFS 38.9% 5-year CSS 40.4%	90-day mortality rate 1.8%
Passaperuma et al., Can J Urol 2006 [41]	Retrospective, single centre	MBC with at least partial response to first-line chemotherapy	12	Consolidative pelvic radiotherapy	Median pelvic-PFS 12.8 months Median OS 15.6 months	No grade 3–4 toxicity
Sweeney et al., J Urol 2003 [42]	Prospective phase II trial	Bladder cancer with retroperitoneal nodal involvement, no visceral metastases, responding to first-line chemotherapy	11	Retroperitoneal lymph node dissection with or without cystectomy	Median PFS 7 months Median CSS 14 months 4-year PFS 27% 4-year CSS 36%	No perioperative mortality

## Combination of radiotherapy and immune checkpoint inhibitors

Immune checkpoint inhibitors (ICI) have been integrated into first-line management of MetBC [3, 45]. Radiation-induced DNA damage causes tumour cell apoptosis, releasing tumour antigens and facilitating immunogenic cell death [46]. Early studies suggest that high-dose radiotherapy or SABR can induce immunostimulatory as well as immunosuppressive changes, leading to unpredictable results when combined with ICI therapy. Tumour irradiation was observed to increase the expression of programmed death ligand 1 (PD-L1) in bladder cancer cells in vitro [47]. On its own, higher PD-L1 is associated with treatment resistance and tumour progression, but PD-L1 blockade by ICIs can synergistically increase tumour cell death. Conversely, late effects of pelvic radiotherapy can be immunosuppressive by selectively decreasing CD4+ T cells and B cells (radiation-induced lymphopenia) and affect response to ICI therapy [46]. In a phase I trial of SABR with immunotherapy for MetBC, tumour response rate was 0% with sequential SABR versus 44% with concurrent pembrolizumab and SABR, suggesting the importance of timing and sequencing of therapies on the immune response [48]. Ongoing phase II ART trial is comparing atezolizumab with or without concurrent SABR in MetBC. Another phase II trial BLAD-RAD01/GETUG-AFU V07 for oligoresidual MetBC stable after first-line chemotherapy is randomising participants to maintenance avelumab alone or with consolidative SABR to bladder and residual metastases. Early experience in non-muscle invasive bladder cancer indicates that determining an appropriate radiotherapy dose in combination with ICI can also be difficult [49]. Dose-limiting urinary toxicity was observed in a phase I trial of pembrolizumab with 36 Gy in 6 weekly fractions to the bladder [50], a radiotherapy dose determined to be safe on its own in an elderly cohort of advanced bladder cancer [19]. Another phase I trial using a lower dose of 18 Gy in 3 fractions with durvalumab observed less adverse effects and considerably reduced efficacy [51]. Delivering an adequately ablative radiotherapy dose to the tumour sites, minimising dose-limiting adverse effects, and establishing optimal sequencing of therapies are major challenges for ongoing and future clinical trials testing radiotherapy-ICI regimens for MetBC.

## Future directions

MetBC is almost universally associated with a dismal prognosis, but its biological heterogeneity is gaining recognition [52, 53]. Within and outside the clinical trials, a proportion of patients show exceptional response to systemic therapies, and eventually survive much longer than expected. These patients should be selected early for intensified treatment to have an opportunity for durable cancer control. Using serum proteomic analysis, antitumour immunity has been suggested as a potential explanation for such exceptional response [54]. Circulating tumour DNA (ctDNA) in the plasma, measurable by a blood test, has shown > 80% mutation concordance with the tumour genome in metastatic urothelial cancer and can be a prognostic biomarker after first-line systemic therapy [52]. Serial assessment of plasma ctDNA can complement tumour biopsy by reflecting mutational changes as the disease progresses, identifying additional targetable mutations [53]. Ongoing trials are collecting biological samples with the aim of discovering reliable biomarkers to identify driver mutations, assess tumour burden, find actionable targets, and monitor treatment response.

## Conclusion

Radiotherapy for metastatic bladder cancer provides effective symptomatic palliation with minimal added toxicity. Advanced treatment techniques have encouraged its evolution as a consolidative therapy to prolong disease control. SABR to metastases and bladder primary, especially for oligometastatic state, is being explored as a feasible and non-invasive ablative treatment. Presently, studies reporting on clinical outcomes with SABR to the bladder primary and/or oligometastases from bladder cancer are retrospective in nature, with small numbers of patients. Randomised trials may be ideal, but impractical for a relatively rare clinical setting of oligometastatic bladder cancer. High-quality evidence may be obtained from innovations in trial designs, such as the OligoRARE phase III basket trial [NCT04498767] randomising patients with oligometastatic cancer to SABR versus palliative radiotherapy, excluding common primaries of prostate, breast, lung, and colorectal cancer [55]. Early clinical trials are also underway to enhance the understanding of the immunomodulatory role of radiotherapy in conjunction with targeted systemic therapies. Prospective evidence from ongoing research will determine the role of radiotherapy in improving clinical outcomes for metastatic bladder cancer.

## Data Availability

This article reports no unpublished data, hence data availability statement is not applicable.
